# Exploring inequalities in access to and use of maternal health services in South Africa

**DOI:** 10.1186/1472-6963-12-120

**Published:** 2012-05-21

**Authors:** Sheetal P Silal, Loveday Penn-Kekana, Bronwyn Harris, Stephen Birch, Diane McIntyre

**Affiliations:** 1 Department of Statistical Sciences, University of Cape Town, Rondebosch, South Africa; 2 Centre for Health Policy, School of Public Health, Faculty of Health Sciences, University of the Witwatersrand, Braamfontein, South Africa; 3 Health Economics Unit, University of Cape Town, Rondebosch, South Africa; 4 Centre for Health Economics and Policy Analysis, McMaster University, Canada; 5 School of Community Based Medicine, University of Manchester, UK

## Abstract

**Background:**

South Africa’s maternal mortality rate (625 deaths/100,000 live births) is high for a middle-income country, although over 90% of pregnant women utilize maternal health services. Alongside HIV/AIDS, barriers to Comprehensive Emergency Obstetric Care currently impede the country’s Millenium Development Goals (MDGs) of reducing child mortality and improving maternal health. While health system barriers to obstetric care have been well documented, “patient-oriented” barriers have been neglected. This article explores affordability, availability and acceptability barriers to obstetric care in South Africa from the perspectives of women who had recently used, or attempted to use, these services.

**Methods:**

A mixed-method study design combined 1,231 quantitative exit interviews with sixteen qualitative in-depth interviews with women (over 18) in two urban and two rural health sub-districts in South Africa. Between June 2008 and September 2009, information was collected on use of, and access to, obstetric services, and socioeconomic and demographic details. Regression analysis was used to test associations between descriptors of the affordability, availability and acceptability of services, and demographic and socioeconomic predictor variables. Qualitative interviews were coded deductively and inductively using ATLAS ti.6. Quantitative and qualitative data were integrated into an analysis of access to obstetric services and related barriers.

**Results:**

Access to obstetric services was impeded by affordability, availability and acceptability barriers. These were unequally distributed, with differences between socioeconomic groups and geographic areas being most important. Rural women faced the greatest barriers, including longest travel times, highest costs associated with delivery, and lowest levels of service acceptability, relative to urban residents. Negative provider-patient interactions, including staff inattentiveness, turning away women in early-labour, shouting at patients, and insensitivity towards those who had experienced stillbirths, also inhibited access and compromised quality of care.

**Conclusions:**

To move towards achieving its MDGs, South Africa cannot just focus on increasing levels of obstetric coverage, but must systematically address the access constraints facing women during pregnancy and delivery. More needs to be done to respond to these “patient-oriented” barriers by improving how and where services are provided, particularly in rural areas and for poor women, as well as altering the attitudes and actions of health care providers.

## Background

Every year there are more than 500 000 maternal deaths worldwide while an estimated 4 million newborns die, and another 3 million babies are stillborn [1]. Most maternal and infant deaths occur in low and middle-income countries, and most are preventable [2]. Globally, most obstetric complications happen around the time of delivery and cannot be predicted [3]. Ensuring skilled attendance at birth is widely acknowledged to be “the single important factor in preventing maternal deaths” [4] and is also recognised as key to reducing stillbirths and neonatal deaths [5]. However it is also recognised that skilled attendance, if it is to really impact on the level of maternal deaths, needs to happen in an “enabling environment” of a well-functioning health care system that provides access to Comprehensive Emergency Obstetric Care (CEOC) including caesarean sections, blood transfusions and other emergency procedures if needed.

A recent review of South Africa’s progress towards achieving the Millenium Development Goals (MDGs) suggests that while substantial progress has been made towards some goals, South Africa will fall well-short on achieving MDGs 4 and 5 (a two-thirds reduction in child mortality rate and a three-quarters reduction in maternal mortality rate) [6]. The reported maternal mortality rate (MMR) in South Africa is contested [7,8]. The latest report on South Africa’s progress in meeting the MDG goals estimates the maternal mortality rate to be 625 deaths per 100,000 live births for 2007; almost double the estimated rate for 2001 of 369 [9]. The Minister of Health recently admitted to parliament that “with regard to curbing child and maternal mortality and improving maternal health, we are in deep trouble” (quoted in [10]).

This MMR is high for a middle-income country, particularly considering that South Africa has high overall reported levels of utilisation of maternal health services with 92% of women reporting one or more ante-natal care (ANC) visit and 91.5% of women having a skilled attendant at delivery [11]. However, these aggregate data hide variations in use by race, urban/rural residence and socioeconomic status - for example in 2003 skilled attendant at delivery for urban women was 94% compared to 85% for rural. Similarly, poor and Black African women were less likely to have a skilled attendant at delivery than their wealthier or white counterparts.

Maternal deaths in South Africa are notifiable and investigated by the National Committee on the Confidential Enquiry into Maternal Deaths (NCCEMD). The NCCEMD (2008) identifies the HIV/AIDS epidemic as one explanation for South Africa’s high MMR, and estimates the institutional MMR for HIV-positive women to be almost ten times that of HIV-negative women. ANC surveillance data estimates that 29% of pregnant women are HIV positive [12]. The NCCEMD report also suggests problems with access to services contributes to maternal deaths . The report identifies patient-orientated factors as contributing to 45.9% of maternal deaths. The two leading patient-orientated factors are a delay in seeking medical help (26.8% of maternal deaths) and not attending or infrequent attendance of ANC (23.7% of maternal deaths) [13].

While health system barriers to access to obstetric care have been fairly well documented in South Africa [6,13], “patient-oriented” barriers to access have been neglected; yet it is crucial to understand, from those needing such services themselves, what these barriers involve. This article therefore explores barriers to access to obstetric care in South Africa from the perspectives of women with needs for care who had recently used, or attempted to use, such care. This is part of a study carried out by a South African/Canadian research collaboration - Researching Equity in Access to Health Care (REACH) - which seeks to explore access barriers to health care in South Africa through three health interventions, viz. maternal health deliveries, tuberculosis care and anti-retroviral therapy for HIV. Access here is defined as the “degree of fit” between the needs of pregnant women during labour and delivery and health system responses. It is comprised of three interlinked dimensions; namely **availability** (e.g. physical location of health facility relative to the population served), **affordability** (e.g. the degree of fit between the health service related costs and the ability of households to meet these costs) and **acceptability** (e.g. providers’ attitudes to and expectations of patients and vice versa). Access represents the *opportunity* or *freedom* to utilise services [14].

## Methods

To explore access to obstetric services from the perspectives of women needing such care, this study drew on mixed methods, sequentially combining quantitative exit interviews (QUAN) with fewer, but detailed, qualitative in-depth interviews (qual) with women who had recently used obstetric services, resulting in a QUAN-qual study design [15].The qualitative phase (Phase 2), while conceptualised at the same time as the quantitative phase (Phase 1), and therefore complementary from the outset, also had the advantage of being conducted after Phase 1 and could be used to triangulate and explore some of the themes emerging from the quantitative data alongside building the larger ‘access’ picture from patient and provider perspectives. Data from both sets of interviews were integrated in the analysis, which sought “elaboration, enhancement, illustration and clarification of the results from one method with results from the other method”; a complementarity approach ( [16] quoted in [17], p.22).

### Quantitative Phase: Sampling methodology and data

Two urban and two rural health sub-districts in South Africa were purposively selected as study sites. The rural sites were chosen because both are Demographic Surveillance System sites and we could draw on existing secondary datasets and contribute to building knowledge in these areas; and the urban sites were selected in large metropolitan areas in consultation with user partners – district managers and local authorities - familiar with sub-districts facing ‘typical’ access challenges. The four sub-districts were extremely geographically dispersed making contamination of information unlikely. An exit interview questionnaire was developed to collect information on the use of, and access to, maternal health services as well as socioeconomic and demographic information. Utilisation details included type of delivery and number of nights spent at the facility, while access information related to the availability, affordability and acceptability of services e.g. travel time to facility, costs incurred and health workers’ attitudes to patients.

A sample size of 300 women per site was calculated based on an anticipated analysis of socio-economic inequalities in use of obstetric health services (*χ*^2^Goodness of Fit test,80 % power, medium effect size). All obstetric health facilities in the rural sub-districts were included, and the number of interviews per facility was proportional to the number of deliveries that took place in those facilities. In the urban sub-districts, obstetric health facilities were selected using the probability proportional to size methodology [18]. The interview included questions on the experience of delivery and therefore was conducted as patients left the facility. All women above the age of 18 who had been discharged from the post-natal ward were eligible for selection. Patients were selected systematically for interview until target sample sizes were reached for all facilities.

### Quantitative Phase Data Analysis

Completed questionnaires and records were entered into EpiData v1.3 and analysed in Stata ®10. To develop an indicator of socioeconomic status (SES) multiple correspondence analysis was conducted on several household level variables including type of house, material of walls, type of toilet, primary source of energy for cooking and ownership of assets such as a vehicle, fridge and livestock etc. The first dimension captured 65% of total inertia and was adopted as the index for socioeconomic status.

Descriptive statistics were calculated. Differences between categorical variables were tested using the Chi-square test of association and differences between ordinal variables were tested using a Chi-square test for Trend. The non-parametric Kruskal Wallis test was used to compare continuous distributions between two groups. Multiple Linear Regression analysis was also used to test associations between descriptors of the affordability, availability and acceptability of services and demographic and socioeconomic predictor variables. Where the dependent variable was categorical, logistic regression was used.

Proxies were used to estimate the availability, affordability and acceptability dimensions of access. The amount of time taken in minutes for the patient to travel to the facility was used as the proxy for availability of the service. If patients travelled to a primary health care facility and needed to be moved to a district hospital by ambulance, then only the travel time from the primary health care facility to the district hospital was recorded. The total amount of money spent on the day of delivery measured as a percentage of annual household expenditure, was the estimator of affordability. This amount comprised money spent on transport, supplies such as sanitary towels and nappies, food, phoning and money used to pay someone for taking over tasks that the patient would be completing such as childcare. South Africa’s legislation prohibit user fees being charged for maternal services at public facilities and none of the sample subjects reported expenditures on medicines. In the logistic regression, the proxy for acceptability was the response to the statement “The health worker is too busy to listen to my problems”. Additionally, other acceptability variables, including levels of respect and whether health workers understood the difficulty of labour were also considered in a separate analysis.

### Qualitative Phase: Sampling methodology and data

A delayed start to the quantitative phase of work in one rural sub-district meant that the qualitative phase could only be completed in three of the four sub-districts. In this phase, in-depth interviews were carried out with women chosen through a purposeful selection methodology to reflect a range of different delivery experiences, and with a particular focus on women who had obviously faced problems accessing services. As this was a facility-located, rather than community-based, sample of women, those who had given birth before they got to the facility, known as Born Before Arrivals (BBAs), were used as a proxy for women who were not able to access services. A total of 16 women who had recently delivered, in most cases at the facility were interviewed shortly after the birth. Follow-up interviews were conducted with six of these women a few weeks later in their own homes. Eight women were BBAs, five had had successful deliveries (three normal vaginal deliveries and two caesarean sections), and three had experienced stillbirths.

Interview guides were loosely structured to explore patients’ life narratives, starting with their latest pregnancy and birth experience, and extending to previous pregnancies and engagements with the health system, as well as their life circumstances and backgrounds more generally. The women were interviewed by trained fieldworkers with previous qualitative research experience and interviews were conducted in the participants’ own language. These were audio-taped, transcribed and translated into English, and pseudonyms were assigned to protect confidentiality. For those women who were interviewed twice, the research team reviewed the initial interview and identified questions (for clarification and/or further exploration) for the follow-up interview. Interviews were independently coded using ATLAS ti.6 by at least two members of the research team. Coding was both deductive, using a codebook constructed around key access issues, and inductive, allowing for themes to emerge from the data.

### QUAN-qual integration Phase

The data and preliminary analyses from both the quantitative and qualitative interviews were then integrated into a broader analysis of access to obstetric services from perspectives of women across and within the study sites. Thematic areas explored in this integrated approach included the availability, affordability and acceptability of obstetric services and barriers to access along each of these dimensions.

### Other methodological issues

The data were collected over a period of 15 months between June 2008 and September 2009. The Universities of Cape Town, Witwatersrand and Kwa-Zulu Natal and the South African Provincial Health Research Committees granted ethical clearance and informed, written consent was obtained from the women for the quantitative exit and in-depth interviews.

## Results

### Patient Characteristics

There was an approximately equal distribution of study participants in urban (51%) and rural (49%) sites with the majority of participants (69% and 100% in urban and rural sites respectively) belonging to the Black African race group (Table 1). Eighteen percent of participants were between 18 and 20 years old with 56% of participants belonging to the 21 to 29 years age category. More participants in rural sites were married or living with a partner than in urban sites (73% vs 69%). The caesarean section rate did not differ significantly between study participants in urban and rural sites (p = 0.71). Of the 1,231 study participants, 1,022 patients had agreed to be tested for HIV during their antenatal care and 276 (27% of those tested) were HIV positive. Among HIV positive participants, 58% were from rural sites and 76% were classified as poor.

**Table 1:** Patient Characteristics

	**Urban n (%)**	**Rural n (%)**	**Statistic (p-value)**
**Socio demographic Variables**			
Study Site:			
Bushbuckridge		299	
Hlabisa		300	
Mitchells Plain	342		
Soweto	290		
Total	632 (51%)	599 (49%)	
**Socio-economic status: (quintiles)**			Independence
1 (Poorest)	14 (2.2%)	233 (38.9%)	*χ*^2^ = 647.1
2	39 (6.2%)	207 (34.6%)	P = 0.000
3	132 (20.9%)	114 (19.0%)	Location
4	207 (32.8%)	39 (6.5%)	*χ*^2^ = 626.5
5 (Richest)	240 (38.0%)	6 (1%)	P = 0.000
Total	632	599	Dispersion
			*χ*^2^ = 0.005
			P = 0.945
**Marital Status :**			
Married/Living with partner	389 (61.6%)	436 (72.8%)	
Single/Divorced/ Widower	243 (38.4%)	163 (27.2%)	*χ*^2^ = 17.57
Total	632	599	P = 0.000
**Race:**			
Black African	437 (69.2%)	598 (100%)	
Coloured	194 (30.7%)		
White	1 (0.7%)		^*χ*2^ = 219.3
Total	632	598	P = 0.000
**Age:**			
18 – 20	87 (13.8%)	135 (22.5%)	
21 – 29	371(58.7%)	315 (52.6%)	
30 – 39	163 (25.8%)	137 (22.9%)	
40 – 49	11 (1.7%)	11 (1.8%)	
Greater than 50	0 (0%)	1 (0.2%)	*χ*^2^ = 17.3
Total	632	599	P = 0.002
**Education**:			
No schooling	5 (0.8%)	17 (2.8%)	
Some schooling	348 (55.2%)	351 (58.6%)	
Matriculation	256 (40.6%)	207 (34.6%)	
Tertiary Education	22 (3.5%)	24 (4.0%)	*χ*^2^ = 11.3
Total	632	599	P = 0.023
**HIV positive:**			
Yes	120 (19.0%)	164 (27.4%)	*χ*^2^ = 24.2
No	470 (74.3%)	324 (54.1%)	P = 0.000
Missing	42 (6.6%)	111 (18.5%)	*χ*^2^ excluding
Total	632	599	missing values
**Type of delivery:**			
Normal Vaginal Delivery	491 (77.7%)	460 (76.8%)	
Caesarean Section	141 (22.3%)	139 (23.2%)	*χ*^2^= 0.14
Total	632	599	P = 0.71

Table 1 provides the demographic characteristics of patient in the sample.

### Availability

Table 2 provides the regression results on proxies for availability, affordability and acceptability of obstetric services while Table 3 provides the reference categories for the independent categorical variables. The total variability explained by predictors in the multiple linear regression model for availability is 30.5%. The model shows significant associations between travel time to the facility and education, age, site and type of facility (p<0.05). Those who completed high school and those who had a tertiary qualification travelled 17 (p=0.02) and 23 (p=0.01) minutes less respectively on average than those without, or with only primary education to get to their facility, holding all other variables constant. Year on year increases in age were associated with a 0.9 minute average drop in travel time holding other variables constant (p=0.006). The reference category for the site variables is Rural1. Patients in site Urban1 took on average 8.5 minutes less to get to the facility than patients in site Rural1 (p=0.049), while those in the second rural site took on average 56 minutes longer than patients in Rural1 holding all variables constant (p<0.001). Differences in average travel time between Rural1 and Urban2 were not statistically significant. Patients who were travelling to primary health care clinics took on average 23 minutes less than those travelling to district hospitals, holding all other variables constant (p<0.001). Travel time was also associated with socioeconomic status, though this result was not significant with a p-value between 0.05 and 0.1. Here, being wealthier was associated with a shorter travel time to delivery facility compared to the poorest quintile.

**Table 2:** Regression Results

	**Access Variables**		
	**Availability**	**Affordability**	**Accessibility**
Predictor variables	Travel Time to facility (minutes)	Total costs incurred on day of delivery as a percentage of Household expenditure	Health worker too busy to listen to my problems (Agree= 1)
	*β*Coefficient (95% C.I.)	*β*Coefficient (95% C.I.)	Odds ratio (95% C.I.)
R^2^	30.5%	14.0%	18.3% (Pseudo)
SES: q2	17.04 (-1.8, 35.8) ^**^	-0.0012 (-0.009, 0.007)	1.47 (0.9, 2.3) ^**^
SES: q3	-11.34 (-24.6, 1.9)^**^	-0.0035(-0.012, 0.005)	1.79 (1.0, 3.1) ^*^
SES: q4	-9.35 (-23.9, 5.18)	-0.0105 (-0.020, -0.001)^*^	0.86 (0.4, 1.8)
SES: q5	-12.76 (-27.1, 1.6) ^**^	-0.0133(-0.023, -0.004)^*^	1.01 (0.5, 2.2)
Marital category: Live with partner	-3.49 (-12.0, 5.0)	0.0026 (-0.005, 0.010)	0.64 (0.3. 1.3)
Marital category: Single	-0.64 (-7.7, 6.4)	0.0005 (-0.004, 0.005)	0.76 (04, 1.3)
Marital category: Widowed/ Divorced/ Separated	-9.95 (-22.5, 2.6)	-0.0061 (-0.014, 0.002)	1.26 (0.2, 9.2)
Sex of Head of Household: Male	1.99 (-5.6, 9.6)	-0.0012 (-0.005, 0.003)	0.94 (0.7, 1.3)
Born in province: yes	-1.59 (-7.4, 4.3)	-0.0005 (-0.005, 0.004)	1.01 (0.6, 1.7)
Education category: Some High School	-13.23 (-26.8, 0.4) ^**^	0.0046 (-0.002, 0.011)	1.38 (0.8, 2.5)
Education category: Completed High School	-17.02 (-30.9, -3.1) ^*^	0.0053(-0.001, 0.012)	1.07 (0.6, 2.0)
Education category: Tertiary qualification	-22.71 (-40.4, -5.0) ^*^	0.0007 (-0.009, 0.011)	0.67 (0.2, 2.8)
Employment status: Employed	5.29 (-5.1, 15.6)	0.0003 (-0.003, 0.004)	1.31 (0.9, 2.0)
Age	-0.88 (-1.5, -0.2) ^*^	-6.26×1^0−06^(-0.0004, 0.0004)	0.98 (0.95, 1.0)
Site: Urban1	-8.48 (-16.9, -0.0) ^*^	-0.0102 (-0.018, -0.003)^*^	0.2 (0.1, 0.4) ^*^
Site: Rural2	55.73 (42.1, 69.3) ^*^	-0.0084 (-0.017, -0.0002)^*^	3.41 (2.1, 5.6) ^*^
Site: Urban2	4.87 (-6.3, 16.1)	-0.0211 (-0.029, -0.013)^*^	0.23 (0.1, 0.5) ^*^
Facility: Primary Healthcare clinic	-23.3 (-29.2, -17.5) ^*^	0.0066 (0.003, 0.010)^*^	3.69 (1.9, 7.0) ^*^
HIV status: positive	0.31 (-9.0, 9.6)	0.0010 (-0.004, 0.006)	1.51 (1.0, 2.2) ^*^
Birth type: Caesar	-	0.0013 (-0.004, 0.006)	0.70 (0.5, 1.1)^**^
Constant	84.31 (59.2, 109.4)^*^	0.0349(0.020, 0.050)^*^	-

Table 2 provides the regression results on proxies for availability, affordability and acceptability.

^*^p-value< 0.05.

^**^0.05≤ p-value ≤ 0.10.

### Affordability

The total variability explained by predictors in the multiple linear regression model for affordability is 14%. The proxy for affordability, total costs incurred on the day of delivery as a percentage of annual household expenditure, is significantly associated with socioeconomic and demographic variables such as the socioeconomic index (SES variable in Table 2), site and delivering at a clinic as opposed to a hospital. Associations with SES show that patients from wealthier households spent significantly less money as a percentage of household expenditure compared to patients from the poorest households holding other variables constant. This result is only significant for households in quintiles 4 (p=0.03) and 5 (p=0.01) and associated coefficients are quite variable as seen from the wide confidence intervals. This result is to be expected as wealthier households should find costs more affordable than poor households. Neither employment nor education yielded significant associations with costs as a percentage of household expenditure. In the urban sites, the costs as a percentage of household expenditure were on average 1% and 2% below that of Rural 1 (p<0.01 for both urban sites). There is also a significant difference between the two rural sites with Rural2 having a 0.8% lower cost as a percentage of household expenditure compared to Rural1 (p=0.045). In these sites, those who delivered at the clinic also had a 0.6% higher cost as a percentage of household expenditure compared to those who delivered at the district hospital, holding all variables constant. This difference in cost is largely attributable to higher average spending on supplies (p=0.004).

### Acceptability

Logistic regression performed on the binary variable “Health worker was too busy to listen to my problem” yielded significant associations with socioeconomic status, site, facility and HIV status. While not all socioeconomic associations were significant, those who fell in quintiles 2 and 3 were more likely to feel that the health worker was too busy than those in quintile 1 (the poorest). Patients in both the urban sites were on average 80% less likely to feel that the health worker was too busy compared to patients in Rural1 (p<0.01), while patients in Rural2 were 3.4 times more likely to feel that the health worker was too busy compared to patients in Rural1 (p<0.01). Those delivering in primary health care facilities were 3.7 times more likely than those delivering at hospitals to feel that the health worker was too busy (p<0.01) and those who were HIV positive were 1.5 times more likely to feel the same way (p=0.03).

**Table 3:** Reference Categories for Regression

1	ES: quintile 1 (poorest)
2	Marital category: Married
3	Sex of head of household: Female
4	Born in Province: No
5	Education category: No/Some/Completed Primary school
6	Employment status: Unemployed
7	Site: Rural 1
8	Facility: District Hospital
9	HIV Status: Negative
10	Birth Type: Normal Vaginal Delivery (NVD)

Table 3 provides a list of the reference categories for the regression analysis.

As acceptability of services is a very broad dimension of access, other proxies are also considered. These include, among others, feeling respected by health workers, being shouted at during labour, having your privacy respected and being able to talk to a doctor privately. These proxies speak to a number of issues regarding patient-staff engagement. Hypothesis test results for other proxies of acceptability in Table 4 show that a greater proportion of participants in the rural sites felt that the health worker was too busy to attend to them compared to those in the urban sites (p<0.001). Respect from the health worker and having your privacy respected was also significantly higher in urban sites than rural sites (p<0.001 and p=0.002 respectively). The rate of being able to talk to doctors privately was low generally; very low in Rural1 and very high in Rural2 (p<0.001). Ethical clearance did not allow for interviews with women under the age of 18 to be interviewed and inequalities in access to obstetric care by age are assessed by comparing participants between 18 and 20 years (18% of sample) with participants over the age of 20. Table 5 shows that while no significant age group differences were found in the proportion of individuals feeling respected by the health worker, participants from the 18 to 20 age group were more satisfied with the service at the facility. While 17% of women reported that health care workers shouted at them during labour, there was no significant difference between the two age groups. The proportion of participants in the 18 to 20 age group agreeing that doctors explained what to expect when giving birth was significantly lower than that of participants older than 20 (p=0.05). Eighty five percent of women in the 18 to 20 age group were in labour for the first time and significantly fewer felt that the health workers understood the difficulty of labour compared to the older participants (p=0.026), 27% of whom were undergoing their first delivery. A significantly greater proportion of HIV positive participants felt that health workers were too busy to attend to them (p=0.000) though there was no significant difference between HIV positive and negative patients in feeling respected by the health worker. A significantly greater proportion (63%) of HIV positive participants felt that they were able to talk to doctors privately compared to HIV negative patients (p<0.001).

**Table 4:** Hypothesis Testing for Acceptability of obstetric services

**Urban – Rural Inequalities**	**Rural1 n(%)**	**Rural2 n(%)**	**Urban1 n(%)**	**Urban2 n(%)**	**Statistic (p-value)**
HW too busy	65(25.6%)	137 (45.7%)	19 (6.6%)	44 (13.6%)	*χ*^2^ = 149.62
Total	254	300	290	323	P = 0.000
HW Respect me	188(62.9%)	197(65.9%)	216 (74.5%)	243 (71.5%)	*χ*^2^ = 11.55
Total	299	299	290	340	P = 0.009
Privacy respected	255(85.6%)	265(88.6%)	275 (95.2%)	300 (88.8%)	*χ*^2^ = 15.12
Total	298	299	289	338	P = 0.002
Talk in private with doctors	87(29.3%)	219(75.8%)	155 (53.5%)	146 (42.7%)	*χ*^2^ = 136.44
Total	297	289	290	342	P = 0.000

Table 4 provide additional results from hypothesis testing on other measures of acceptability.

**Table 5:** Hypothesis Testing for Acceptability of obstetric services

**Age related Inequalities**	**18 – 20 years n (%)**	**Greater than 20 years n (%)**	**Statistic (p-value)**
HW Respect me	148 (66.67%)	696(69.25%)	*χ*^2^ = 0.556
Total	222	1005	P = 0.452
Satistfied with service	212 (95.5%)	917 (91.24%)	*χ*^2^ = 4.47
Total	222	1005	P = 0.034
Shouted at during labour	34 (15.32%)	174 (17.38%)	*χ*^2^ = 0.55
Total	222	1001	P = 0.458
Expectations of giving birth were discussed	161 (72.85%)	790 (79.16%)	*χ*^2^ = 5.92
Total	221	998	P = 0.05
HW understood the difficulty of labour	191 (86.82%)	918 (91.62%)	*χ*^2^ = 4.95
Total	220	1002	P = 0.026
**HIV status related Inequalities**	**HIV positive n (%)**	**HIV negative n (%)**	**Statistic (p-value)**
HW Respect me	194 (68.31%)	549 (69.49%)	*χ*^2^ = 0.1373
Total	284	790	P = 0.711
HW too busy	84 (30.43%)	139 (18.41%)	*χ*^2^ = 17.24
Total	276	755	P = 0.000
HW understood the difficulty of labour	259 (91.52%)	712 (90.47%)	*χ*^2^ = 0.273
Total	283	787	P = 0.601
Talk in private with doctors	175 (62.50%)	372 (47.21%)	*χ*^2^ = 19.33
Total	280	788	P = 0.000

Table 5 provide additional results from hypothesis testing on other measures of acceptability.

### Access constraints faced by women who delivered before arriving at a facility

The qualitative data highlight the range of availability, affordability and acceptability constraints faced by pregnant women, particularly for the eight women who had delivered before they arrived at a health facility. While a relatively heterogeneous group, with a range of different reasons for not giving birth in a facility, all had originally intended to deliver at a facility - although two had given up on this intention due to poor interactions with the health service during ANC or early in labour.

One woman who “gave up” had been in denial about her pregnancy, which she kept secret. She had struggled to access services during ANC and once had been turned away because she went “on the wrong day”, with the financial implications being too costly to contemplate returning. Based largely on hearsay, she had feared that health care workers would “judge and shout” at her as she was young, poverty-stricken and still going to school. She told herself, “If it takes for me to give birth by myself so be it because it seems like I don’t have a choice.... . I told myself I have been through a lot more than this, big thing ........ and this is just a temporary pain and it will pass”. After delivering under a tree at home she was taken to the clinic by ambulance and was treated well, reflecting that “the people were very friendly, they didn’t judge me in the way that I thought they would so they talked nicely actually...... I feel like I should have went to the clinic and not listened to the people...... they were not as bad as I thought or as I was told they were”.

Another woman “gave up” on delivering within a facility after she had been to a clinic but was told she was only in early labour and to come back later. She pleaded with the nurses that she lived far from the clinic and that it would be hard for her to come back but she was told the clinic was full. She then went home and delivered with the help of a neighbour who was experienced in giving massage to assist women with deliveries.

Four of the women delivered at home waiting for transport (mainly ambulances), and two delivered on the way to the facility. Ambulances in five of the eight cases arrived too late for the delivery but transported the women and the new-born baby to hospital. One woman, who was having her fifth child, lived in an informal settlement and the ambulance service said it would come to the nearest clinic, which was still some distance from her house. She did not have funds to organise other forms of transport. The ambulance took four hours to get to the clinic by which time she had given birth. Her boyfriend then met the ambulance at the clinic and directed it to the shack where they lived and it picked up the woman and baby and took them to the nearest clinic. Another woman who, having her third child, lived in a hostel, and did not have funds for other forms of transport, was also told that the ambulance would not come into the area where she lived. She was in too much pain to walk to the ambulance waiting at the gates of the compound and so gave birth in her room. The ambulance then took her and the baby to hospital.

Other women who gave birth before reaching a facility tried to organise private transport - with the help of partners or family - when they realised that the ambulance would take a while or not arrive. Some neighbours helped out by transporting them for small amounts of money (often to be paid back later), while other neighbours or a community member with a car, charged large amounts, sometimes as high as US$55.

Another woman reported that she had started labour in the night but not been able to organise transport until 6am in the morning. She had got to the clinic but because she was in premature labour the nurses called an ambulance to transfer her to the hospital, telling her “we can do nothing for you here”. The ambulance came quickly but she then gave birth a few seconds after walking to the ambulance. The ambulance driver helped her and was nice, in contrast to the nurses who remained disengaged, but the baby was stillborn. When she arrived at the hospital she was told sternly that she should have stayed at the clinic. She was then transferred to a bed and the baby placed on the floor next to her in a plastic bag.

### Qualitative findings on acceptability of services

The qualitative interviews painted a different picture of the acceptability of the services to that emerging from the quantitative component of the study. All but one of the women interviewed expressed dissatisfaction with the quality of care that they received both during ANC and during the delivery - although in most cases there were some health care workers and aspects of their care they had been happy with. Most women recognised the difficult conditions that health care providers worked under, but still felt that they deserved to receive better care than they did. As one woman who had a stillbirth stated: “I understand that they are overworked and underpaid but it is not my problem. It should not affect my health”. Complaints about the quality of care that women received included nurses “hurling insults at them”, mocking women who did not understand what they were saying, “if you walked a little bit slow then they scream at you in front of the people”, and not being patient or sympathetic to the pain that they were in. The four women who had stillbirths, perhaps not surprisingly due to their loss, were particularly upset about the treatment that they received. As well as echoing the complaints of all women about the overall quality of care, they were particularly upset about how they were treated after their babies had died. They felt that the way that they learned their baby had died was not good, often hearing it from health care workers talking about it among themselves and not directly. They also complained about how, after their stillbirths, they were placed in wards with women with live babies. As one woman said “I could have been better off if they took me to a room for the mentally ill people rather than in a room where there were people carrying their babies and I stayed there and I was crying cause babies were crying and I could not take it you know”.

## Discussion

Health and health service delivery have been affected by relics of South Africa’s past including racial and gender discrimination, violence and severe income inequalities [19]. The government that came to power in 1994 took several steps to address inequalities in health services and to improve access, particularly in terms of availability and affordability. For example, more than 1,300 primary health care clinics were constructed in areas that were under-served [20]. Our findings highlight the importance of this initiative in improving service availability in relation to geographic access; the average travelling time to clinics was 23 minutes less than to hospitals. Nevertheless, travelling times can be high in rural areas (even to clinics) and accessing transport to a health facility can be problematic. This is highlighted in the qualitative findings where most women who delivered before arrival at a facility did so while waiting for transport, particularly ambulances. Importantly, this is not only a problem in rural areas but also in urban informal settlements where ambulances sometimes refuse to go. Thus making obsetric care more available involves more than just the number and proximity of facilities.

Another initiative of the first democratically-elected government was the removal of user fees for all services for pregnant women and children under-six years at public sector facilities. This served to reduce the financial barriers to accessing delivery, and other maternal and child health services. However, our findings indicate that some affordability barriers remain, as patients still have to bear costs, which can sometimes be quite high, for transport to reach facilities and to purchase supplies required for the delivery. This is particularly true for rural areas. The qualitative data highlights that transport costs in particular create a barrier to access when public transport is not accessible (e.g. at night) and there are difficulties in getting ambulances; the costs of private transport can be beyond the reach of many households. It is important to recognise that there is sometimes a trade-off between availability and affordability; while clinics are more geographically accessible than hospitals, costs to patients at clinics are higher than at hospitals largely due to the greater need to purchase supplies for a delivery in clinics. Acceptability of services is also perceived to be worse at the clinic level.

Despite the policy efforts to improve availability and affordability of health care, there have been severe problems with implementation of some of these policies, as well as with the training, distribution and motivation of health care workers [20]. Indeed, some of the policies, particularly the free care for pregnant women and young children, contributed to declining staff morale as it was introduced without engagement with frontline health workers and increased staff workloads as demand increased without corresponding increases in real resources to meet those demands [21,22]. This is particularly true for rural areas. As our results show, the acceptability dimension represents a barrier to service access. The quantitative results highlight how a greater proportion of patients in rural sites felt that the health worker was too busy. The same was felt for HIV positive patients across all four sites. The qualitative results highlight how one bad experience of health services (e.g. being turned away from ANC services due to coming on the “wrong day”) can translate into not wanting to return to a health facility for delivery. Poor staff engagements with patients range from shouting at patients during labour (reported by 17% of patients) to highly insensitive behaviour towards patients who had experienced stillbirths.

Although South Africa has high levels of maternal health service utilisation, with over 90% of pregnant women attending ANC and having a skilled attendant at delivery, our study demonstrates that many women still face considerable access barriers. Rural women face the greatest access barriers, such as experiencing the longest travel times, the highest costs associated with delivery and lowest service acceptability relative to women living in urban areas. Although sometimes not statistically significant, there was also a strong socioeconomic relationship with worse access for poorer women, particularly in terms of the availability and acceptability dimensions. Although focusing on utilisation of maternal health services rather than access issues, Say and Raine (2007) [23], Houweling (2007) [24], and Gabrysch and Campbell (2009) [3] similarly noted inequalities in favour of wealthier women and urban dwellers. Acharya (2009) [25] also found that rural women had difficulty in accessing emergency obstetric care in district hospitals owing to barriers like distance, cost of transport, shortage of medicines at the hospital and problems with staff attitude towards the poor.

High levels of deliveries attended by skilled health workers are not enough to prevent maternal mortality. Lengthy travel times to facilities and delays in securing transport contribute to maternal mortality. Studies in parts of India found that between 42% and 52% of maternal deaths occurred at home or in transit to the hospital [26]. Further, as noted by Thaddeus and Maine (1994), getting to a health facility is only one element of the access challenge; getting good quality of care at the facility is entirely another issue [27]. Poor provider-patient interactions, including inattentiveness of staff to a patient’s condition and turning women away due to not being “ready” to deliver, can also contribute to avoidable maternal deaths.

In order to move towards the MDGs, it is essential to focus not simply on increasing maternal service coverage levels, but to address systematically all of the access constraints that pregnant women face. As noted by Gabrysch and Campbell (2009, p1) [3], “it is important to consider as many influential factors as possible in any analysis of delivery service use”.

### Limitations of study

This study was facility-based and therefore only interviewed women who had used services, either at delivery or soon after delivery and hence had overcome any barriers to access to services. Although a household survey would potentially provide a more representative sample of the populations of the study sites this was not possible for the current study. We are therefore analysing the size and nature of barriers to access among this group of facility ‘users’ in order to assess the distribution of these factors within and between the study populations. We do not know the stories or experiences of women who did not use the health care facilities studied and hence the size and nature of access barriers among non-users may be very different to those identified in this study. The two rural areas in this study are also demographic surveillance sites with previous work suggesting high levels of home births. Clearly decisions about home births may be influenced by barriers to access to health care facilities. We are undertaking a utilisation incidence analysis to compare the population of women who used facility-based obstetric services with those who did not use these services.

We attempted to collect information on all aspects of the opportunity cost of using the service covering both direct out of pocket costs (transport fares, supplies) and indirect costs (time travelling to and waiting at the facility). However we did not collect information on informal payments to providers (‘bribes’) because these are not generally considered to be a feature of the public health care system in South Africa. The interview used to collect information on patient costs, specifically asked for the costs of getting to the first facility attended. So, for women who went first to a clinic but were then transferred to a hospital, we did not have information on any out of pocket costs associated with this transfer. The reason for this was twofold: firstly, most transfers are done by ambulance and the second reason was to avoid the interview becoming too long. As a result our estimate of costs is conservative. Further the interview used to collect information on patient travel specifically asked for travel time from home to the primary facility and in the case where a transfer was required, travel time from the primary facility to the secondary facility only. As a result our estimate of travel time is also conservative.

Finally, we selected one variable for each dimension of accessibility to study. The findings may have been different for other variables in each dimension. For example, affordability may be associated with the source of funds to meet the costs of receiving care (e.g., household expenditures, borrowing, selling assets). However, the analysis of all aspects of each accessibility dimension is the beyond the scope of this paper.

## Conclusion

Although most women in South Africa do access obstetric care services, this study shows that these services can be unaffordable, unavailable and/or unacceptable for many women, creating barriers that impact on how much, and when, women access them. There are also inequalities in the distribution of these access barriers, with differences between socio-economic groups and geographic areas being the most important. If South Africa wants to ensure that all women use antenatal and obstetric services, and present earlier and more regularly during pregnancy, and timeously in labour, then more needs to be done to respond to “patient-oriented” barriers; the factors that impede the opportunity or freedom of women to use these needed services. This requires improving the “fit” between the health care system and women through improving how and where services are provided, particularly in rural areas and for poor women. It also requires tackling the ways in which services are delivered through the attitudes and actions of health care providers. Taking on board the perceptions and experiences of those most affected - the women themselves - offers the health care system an opportunity to expand beyond simply providing technical, medical care to creating a truly “enabling environment” in which to work towards the Millenium Development Goals.

## Abbreviations

(ANC), Ante-natal care; (BBA), Born Before Arrival; (CEOC), Comprehensive Emergency Obstetric Care; (HW), Health Worker; (MDGs), Millennium Development Goals; (MMR), Maternal Mortality Rate; (NCCEMD), National Committee on the Confidential Enquiry into Maternal Deaths; (SES), Socioeconomic status.

## Competing interests

The authors declare that they have no competing interests.

## Author’s contributions

All authors contributed to the conceptualisation of the research and drafted the article. In addition, SPS undertook the preparation of dataset, interpretation of the data and the statistical analyses, LPK and BH undertook the preparation of the qualitative data and LPK undertook the qualitative data analysis. All authors read and approved the final manuscript.

## Pre-publication history

The pre-publication history for this paper can be accessed here:

http://www.biomedcentral.com/1472-6963/12/120/prepub
